# The relationship between glutathione levels in leukocytes and ocular clinical parameters in glaucoma

**DOI:** 10.1371/journal.pone.0227078

**Published:** 2019-12-30

**Authors:** Takeshi Yabana, Kota Sato, Yukihiro Shiga, Noriko Himori, Kazuko Omodaka, Toru Nakazawa

**Affiliations:** 1 Department of Ophthalmology, Tohoku University Graduate School of Medicine, Sendai, Japan; 2 Collaborative Program for Ophthalmic Drug Discovery, Tohoku University Graduate School of Medicine, Sendai, Japan; 3 Department of Ophthalmic Imaging and Information Analytics, Tohoku University Graduate School of Medicine, Sendai, Japan; 4 Department of Retinal Disease Control, Tohoku University Graduate School of Medicine, Sendai, Japan; 5 Department of Advanced Ophthalmic Medicine, Tohoku University Graduate School of Medicine, Sendai, Japan; Massachusetts Eye & Ear Infirmary, Harvard Medical School, UNITED STATES

## Abstract

**Purpose:**

To investigate the effect of mitochondrial dysfunction on the autoregulation of blood flow, by measuring levels of glutathione, an indicator of mitochondrial dysfunction, in glaucoma patients.

**Methods:**

Fifty-six OAG patients and 21 age-matched controls underwent a blood assay. Mitochondrial function was measured according to the levels of total glutathione (t-GSH), reduced GSH (GSH), and oxidized GSH (GSSG, glutathione disulfide) in peripheral blood mononuclear cells. Ocular blood flow in the optic nerve head was assessed with laser speckle flowgraphy parameters, including acceleration time index (ATI). We determined correlations between these measurements and other clinical parameters. Furthermore, we investigated the association between glutathione levels and glaucoma with a logistic regression analysis. Finally, we calculated the area under the receiver operating characteristic (ROC) curve in order to determine the power of redox index (the log GSH/GSSG ratio) to distinguish the groups.

**Results:**

OAG patients demonstrated significantly higher GSSG levels and a lower redox index than the controls (p = 0.01, p = 0.01, respectively), but total GSH and reduced GSH levels were similar in the OAG subjects and controls (p = 0.80, p = 0.94, respectively). Additionally, redox index was significantly correlated with mean deviation (MD) of the visual field (r = 0.29, p = 0.03) and ATI (r = -0.30, p = 0.03). Multiple linear regression analysis showed that redox index contributed to MD (p = 0.02) and ATI (p = 0.04). The receiver operating characteristic curve (AUC) analysis suggested that redox index could differentiate between control eyes and eyes with glaucoma (AUC; 0.70: 95% interval; 0.57–0.84). The cutoff point for redox index to maximize its sensitivity and specificity was 2.0 (sensitivity: 91.1%, specificity: 42.9%).

**Conclusions:**

These results suggest that redox index is lower in OAG patients than in controls. Thus, it is possible that mitochondrial dysfunction contributes to glaucoma pathogenesis by causing vascular alterations.

## Introduction

Glaucoma is the second most common cause of blindness worldwide; it is characterized by retinal ganglion cell (RGC) death and irreversible visual loss.[1] Aging and increased intraocular pressure (IOP) are glaucoma risk factors, but patients sometimes progress despite having low IOP. This implies that additional causative factors underlie the disease.[2] Increasing evidence suggests that mitochondrial dysfunction plays an important role in the pathology of various neurodegenerative diseases, including Parkinson’s disease,[3] Alzheimer’s disease,[4] Huntington disease,[5] and amyotrophic lateral sclerosis,[6] in addition to glaucoma.[7–14] Mitochondrial dysfunction has two detrimental consequences: excessive reactive oxygen species (ROS) production and decreased ATP synthesis, which together induce RGC loss.[10] The high density of mitochondria in the optic nerve head (ONH) suggests that mitochondrial function is particularly important in this location, and that RGCs are particularly vulnerable to mitochondrial dysfunction.[15, 16]

Glutathione (GSH), a tripeptide consisting of glycine, cysteine and glutamic acid, prevents the effect of ROS when it changes its formation from reduced GSH to oxidized GSH (GSSG, glutathione disulfide).[17] Normally, almost all glutathione in the mitochondria is reduced GSH, but in the presence of mitochondrial dysfunction, the proportion of GSSG increases due to the increased elimination of ROS. Therefore, the GSH/GSSG ratio is an indicator of mitochondrial function.[18] Moreover, previous reports have shown that mitochondrial dysfunction occurs in leukocytes due to systemic mitochondrial failure in diabetes,[19] cancer,[20] and neurodegenerative diseases.[21–23] Additionally, it is thought that GSH is involved in the maintenance of vascular endothelial function, implying that aberrant GSH metabolism could contribute to the pathogenesis of vascular diseases, including cardiovascular disease[24, 25] and stroke.[26, 27] These previous findings suggest that the investigation of GSH levels in leukocytes could also be useful for translational research in glaucoma. Although there have been a number of research projects investigating the relationship between glaucoma and systemic mitochondrial dysfunction, the mechanism underlying this relationship is still unclear.[7, 9, 11] In this study, we examined the relationship between mitochondrial dysfunction, ocular hemodynamics and glaucoma by focusing on GSH levels in the leukocytes of glaucoma patients.

## Materials and methods

### Subjects

Fifty-six OAG patients and 21 age-matched controls were recruited from Tohoku University Hospital, Miyagi, Japan, between December 2016 and February 2017. The procedures in this study followed the tenets of the Declaration of Helsinki and were approved by the institutional review board of Tohoku University Graduate School of Medicine. Written informed consent was obtained from all participants at the time of blood sample collection. Control subjects were recruited from patients who visited Tohoku University Hospital for treatment of diseases other than glaucoma, such as cataract, epiretinal membrane (ERM), dry eye, and traumatic optic neuropathy. No control subject had any history of elevated IOP or any apparent glaucoma. We selected the healthy contralateral eye of each control subject for inclusion. If both eyes had cataract or ERM, we chose the eye with better visual acuity. This study’s ocular exclusion criteria included narrow iridocorneal angles, evidence of secondary open-angle glaucoma, and other non-glaucomatous ocular diseases, including diabetic retinopathy, proliferative vitreoretinopathy, and age-related macular degeneration. We also excluded patients with a history of any chronic systemic disease with presumed abnormal circulating glutathione levels, including cancer,[28] neurodegenerative diseases,[29] autoimmune disease,[30] liver disease,[31] and hematologic diseases.[20] Furthermore, cases for which the gathered quantity of lymphocytes was insufficient to measure GSSG were also excluded.

### Measurement of clinical parameters

All subjects underwent a baseline examination, including best-corrected visual acuity measurements (log MAR: logarithm of the minimal angle of resolution), measurement of axial length (AL) with the Zeiss IOL Master (Carl Zeiss AG, Oberkochen, Germany), slit lamp examination, funduscopic examination, gonioscopy, IOP measurement, optical coherence tomography (3D-OCT 2000; Topcon Corporation, Tokyo, Japan), visual field testing and measurement of systemic variables. IOP was measured with Goldmann applanation tonometry. Circumpapillary retinal nerve fiber layer thickness (cpRNFLT) was measured with spectral-domain OCT. The visual field was analyzed with the 24–2 Swedish interactive threshold algorithm standard program of the Humphrey Field Analyzer (Carl Zeiss Meditec, Dublin, CA, USA). The systemic blood pressure (BP), and heart rate (HR) were recorded in the left brachial artery at the height of the heart with an automated BP monitor (HEM-759E; Omron Corporation, Kyoto, Japan) with the subject in a sitting position. Mean arterial blood pressure (MAP) and ocular perfusion pressure (OPP) were calculated as follows: MAP = diastolic BP + 1/3 (systolic BP–diastolic BP), and OPP = 2/3 MAP–IOP.[32]

### Laser speckle flowgraphy measurement

**Fig 1:**
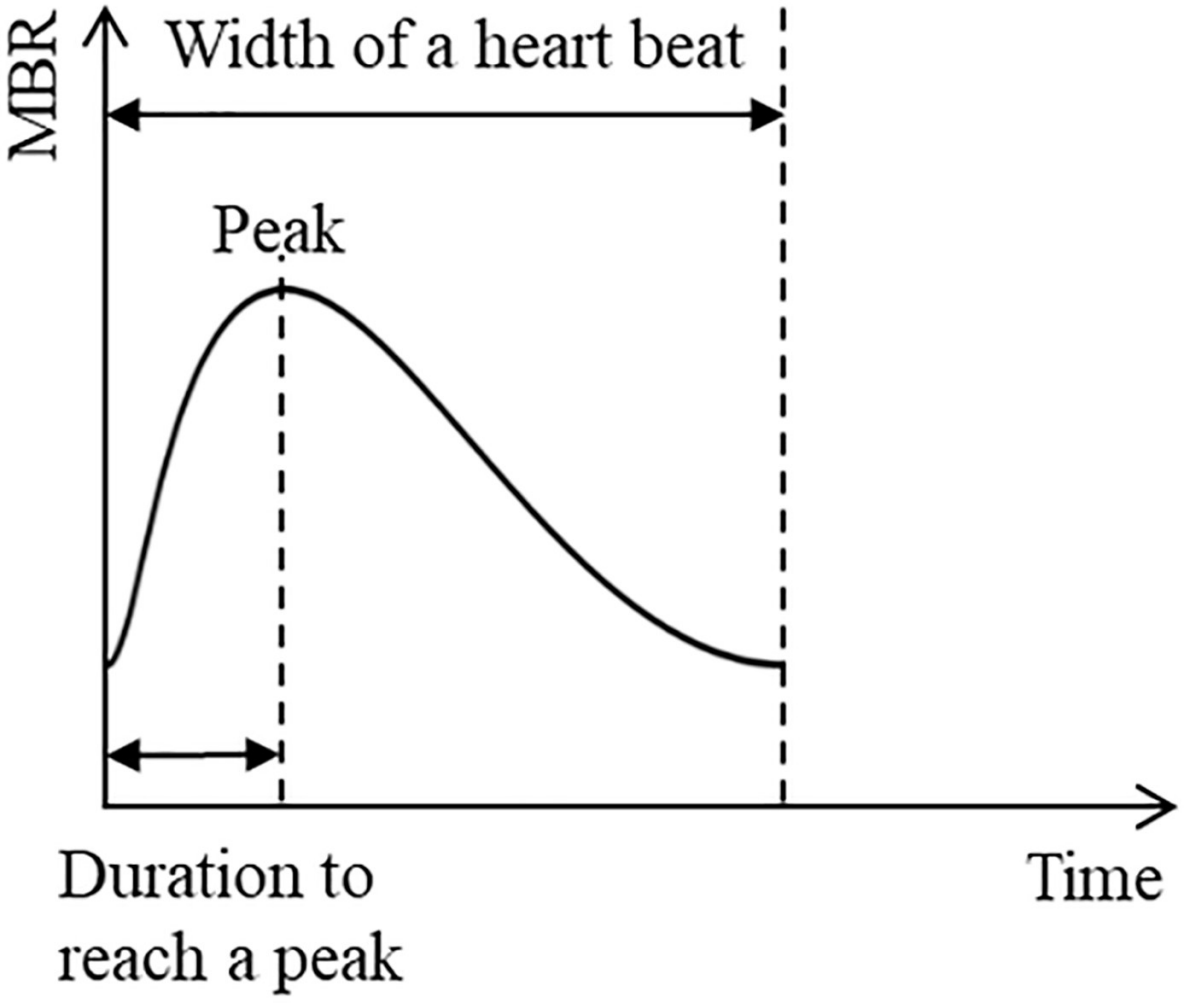
Waveform analyses of acceleration time index (ATI).

The principles of laser speckle flowgraphy (LSFG; Softcare Ltd, Iizuka, Japan) have been described in detail previously.[33] In brief, LSFG allows for noninvasive quantitative estimation of microcirculation in the ONH, the choroid at the fovea, and the retinal vessels, using the laser speckle phenomenon. The LSFG device consists of a fundus camera equipped with a single-mode diode laser emitting light at a wavelength of 830 nm and a digital charge-coupled device camera with 750 x 360 pixels. Before LSFG measurement, the right pupil of each patient was dilated with 0.5% tropicamide and 0.5% phenylephrine hydrochloride. Systolic and diastolic BP and IOP were measured after the subjects had rested for 5 min in a sitting position in a dark room. The mean blur rate (MBR), calculated from variations in the blurring produced by the interference of a laser scattered by blood cells moving in the ocular fundus, is a quantitative index of the relative blood flow (BF) velocity. MBR images are acquired continuously at the rate of 30 frames per second over a 4-second period. In-built software (LSFG Analyzer, Version 3.0.43.0; Softcare Co., Ltd.) then synchronizes all captured MBR images with each cardiac cycle, and the averaged MBR of a heartbeat is displayed as a heartbeat map. The software next divides the MBR in the ONH into the large vessel and capillary areas automatically. In this study, we examined waveform changes only in the capillary area of the ONH because of its reported usefulness in intergroup comparisons.[34] The last step of the analysis was to calculate the acceleration time index (ATI) of the MBR waveform as it changed over the course of a single heartbeat (Fig 1).[35] The ATI is defined as the duration to reach the peak of MBR, and the value is calculated according to the following formula: ATI = (duration to reach peak/duration of a heartbeat) x 100. All statistical analyses of ONH circulation were based on the average of three separate LSFG measurements.

### Glutathione assay

#### Sample collection and extraction of glutathione in mononuclear cells

A total of 10 ml of blood was collected from each subject in an EDTA tube to prevent oxidation and the effects of transition metals such as Fe^2+^. In order to avoid variations and GSH loss, all equipment and samples were maintained at 4° C in ice, including the GSH assay, and pH was maintained in the acidic range.[36] Blood samples were collected at least 3 hours after the last meal.[37] Less than 30 minutes elapsed between blood extraction and the isolation of peripheral blood mononuclear cells (PBMCs).

Each sample was half-diluted with cold serine and poured into a Lymphoprep tube (Axis-Shield, Oslo, Norway). PBMCs were isolated by gradient centrifugation at 800 g for 20 minutes at 4° C. The PBMC layer was collected with a pasteur pipette and suspended in cold serine to decrease the viscosity of the ficoll (the total number of cells in this suspension was calculated with a cell counter). Isolated PBMCs were pelleted by centrifugation (10 min at 1500 g, 4° C). The pellet was disrupted by the addition of 80 μl of 10 mmol/L HCl and cryopulverization twice. In order to stop protein reactions, 40 μl of 5% 5-sulphosalicylic acid (SSA) was added to the sample.[36] The cytosolic extract was cleared by centrifugation (10 min at 8000 g, 4°C). Finally, the volume of the sample was increased to a total of 500 μl with 0.5% SSA. After that, all samples were stored in liquid nitrogen at -196°C until the glutathione assay was performed.

#### Measurement of glutathione levels

The total levels of GSH (t-GSH) and GSSG were assessed with a glutathione reductase-DTNB (5,5’-dithobis-2-nitrobenzoic acid) recycling assay using a GSH/GSSG Quantification kit (Dojindo Laboratories, Kumamoto, Japan). This assay is based on the continued enzymatic regeneration of reduced GSH from the reduction of GSSG by enzyme glutathione reductase and the GSH-mediated reduction of DTNB to a chromogenic product, as described in previous studies.[36, 38, 39] The absorbance of GSH and GSSG were read at 405 nm using a microtiter-plate ELISA reader.[39] Each sample was analyzed in 3 pairs of wells. Finally, the GSH level ([GSH] = [t-GSH]– 2x[GSSG]) and redox index (defined as the logarithm of GSH/GSSG ratio) were calculated. Glutathione concentrations were expressed as nmol/5x10^6^ cells.[40]

### Statistical analysis

All data are presented as the mean ± standard deviation. Differences between groups were assessed with the Mann-Whitney U test. Fisher’s exact test was used to analyze frequency data for sex distribution, diabetes mellitus, hyperlipidemia, hypertension, heart disease, sleep apnea, a smoking habit, and glaucoma family history. Spearman’s rank correlation test was used to evaluate single correlations between GSH measurements and other parameters. Multiple linear regression analysis was performed to determine independent variables affecting mean deviation (MD) and ATI. A receiver operating characteristic (ROC) curve for redox index was plotted to determine the optimum cutoff point, and the area under the receiver operating characteristic curve (AUC) was used to determine the power of redox index to discriminate between control and glaucoma eyes. All statistical analysis was performed with JMP Pro 12 (SAS Institute Japan, Inc., Tokyo, Japan). The significance level was set at p<0.05.

## Results

The characteristics of the study groups are given in Table 1. There were no significant differences (p>0.05) between the glaucoma patients and control subjects in age, gender, AL, IOP, OPP, systemic diseases, systolic BP, diastolic BP, HR, or smoking habits; however, there were significant differences in family history of glaucoma (p = 0.03).

**Table 1:** Baseline characteristics between the control and glaucoma groups.

Characteristics	Control			Glaucoma			p Value
N		21			56		
Age (years)	61.19	±	7.28	58.92	±	8.99	0.31*
Gender (male: female)	11	:	10	29	:	27	1.0^†^
VA (logMAR)	0.01	±	0.29	0.07	±	0.24	0.17*
AL (mm)	25.33	±	1.77	25.46	±	1.52	0.61*
IOP (mmHg)	14.86	±	3.78	13.57	±	3.18	0.11*
CpRNFLT (μm)		-		79.22	±	14.50	-
MD (dB)		-		-14.16	±	6.52	-
MBR (AU)		-		8.76	±	2.23	-
ATI (AU)		-		32.77	±	4.46	-
Systolic blood pressure (mmHg)	130.04	±	15.71	126.27	±	13.95	0.40*
Diastolic blood pressure (mmHg)	82.43	±	12.50	78.95	±	12.04	0.15*
HR (beats/min)	78.14	±	10.35	71.98	±	11.99	0.06*
OPP (mmHg)	53.42	±	7.45	52.31	±	7.87	0.38*
Diabetes mellitus (n)		2			7		1.0^†^
Hyperlipidemia (n)		8			12		0.15^†^
Hypertension (n)		7			16		0.78^†^
Heart disease (n)		3			9		1.0^†^
Sleep apnea		0			5		0.32^†^
Smoking habit		10			20		0.43^†^
Glaucoma family history		0			12		0.03^†^

*Mann-Whitney U test.

†Fischer’s exact test.

VA, visual acuity; log MAR, logarithm of minimum angle of resolution; AL, axial length; IOP, intraocular pressure; CpRNFLT, circumpapillary retinal nerve fiber layer thickness; MD, mean deviation; MBR, mean blur rate; AU, arbitrary unit; ATI, acceleration time index; HR, heart rate; OPP, ocular perfusion pressure.

**Fig 2:**
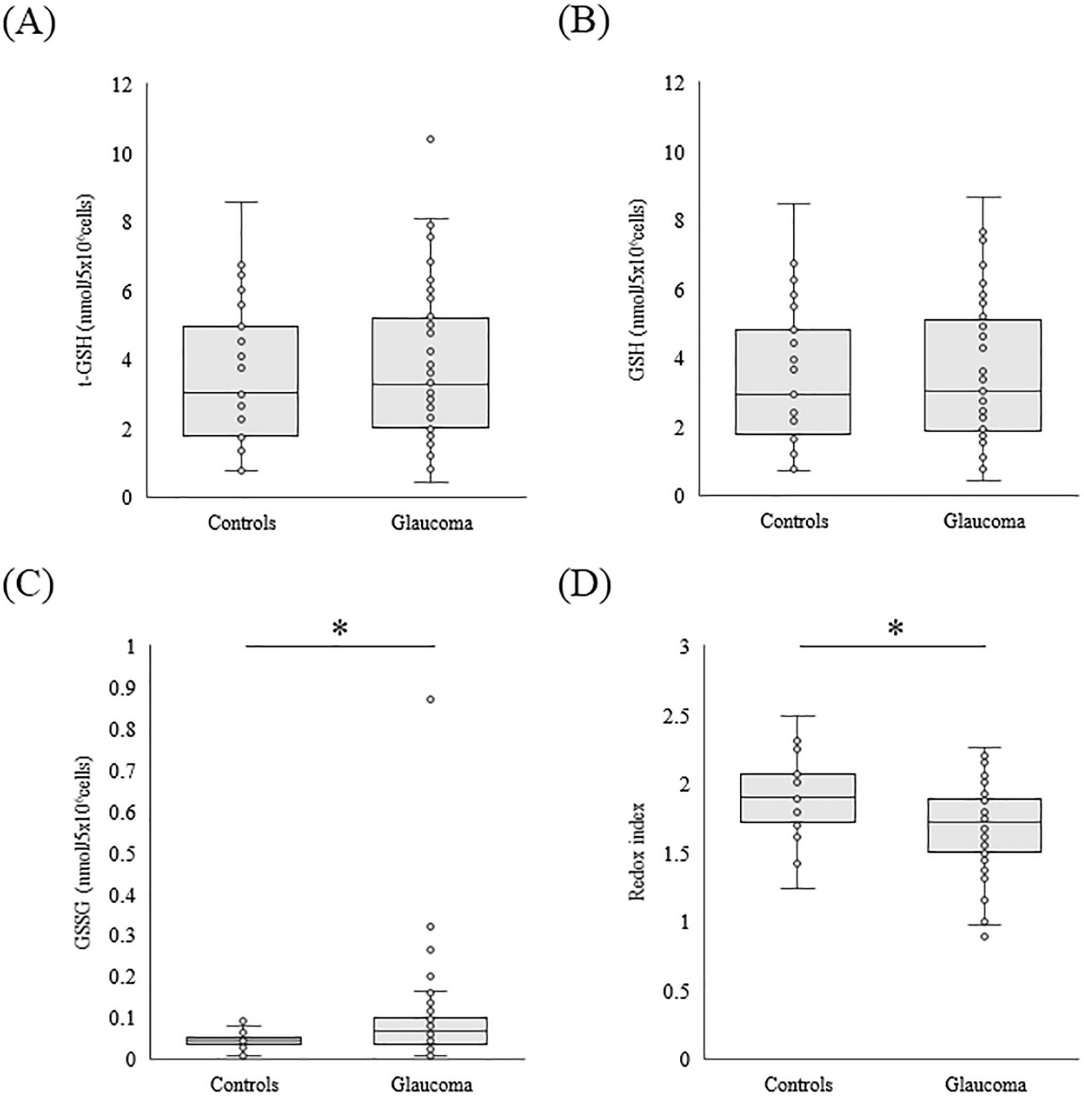
Decreased GSH/GSSG redox balance in glaucoma monocytes. Box plots of t-GSH (A), GSH (B), GSSG (C), and redox index (D) showing differences between the study groups. The bottom and top of the boxes represent the first (25th percentile) and third quartiles (75th percentile), respectively, while the lines inside the boxes indicate the second quartile (50th percentile). The ends of the whiskers represent the 10th and 90th percentiles. Outlying values are included as small circles. The asterisks indicate statistically significant differences between glaucoma patients and controls (Mann-Whitney U test, *p<0.05). t-GSH, total glutathione; GSH, glutathione; GSSG, glutathione disulfide; Redox index, the log GSH/GSSG ratio.

Table 2 shows the GSH and GSSG levels in PBMCs from both groups. The glaucoma patients showed a significantly higher level of GSSG (p = 0.01, Fig 2C) and lower redox index than the control group (p = 0.01, Fig 2D). However, there were no significant differences between the glaucoma patients and controls in t-GSH (p = 0.80, Fig 2A) or GSH (p = 0.94, Fig 2B).

**Table 2:** Glutathione levels in monocytes in the study groups.

	Control			Glaucoma			p Value
t-GSH (nmol/5x10^6^ cells)	3.57	±	2.20	3.73	±	2.19	0.80*
GSH (nmol/5x10^6^ cells)	3.49	±	2.20	3.54	±	2.10	0.94*
GSSG (nmol/5x10^6^ cells)	0.04	±	0.02	0.09	±	0.12	0.01*
Redox index	1.90	±	0.30	1.67	±	0.30	0.01*

*Mann-Whitney U test.

t-GSH, total glutathione; GSH, glutathione; GSSG, glutathione disulfide; redox index, the log GSH/GSSG ratio.

**Fig 3:**
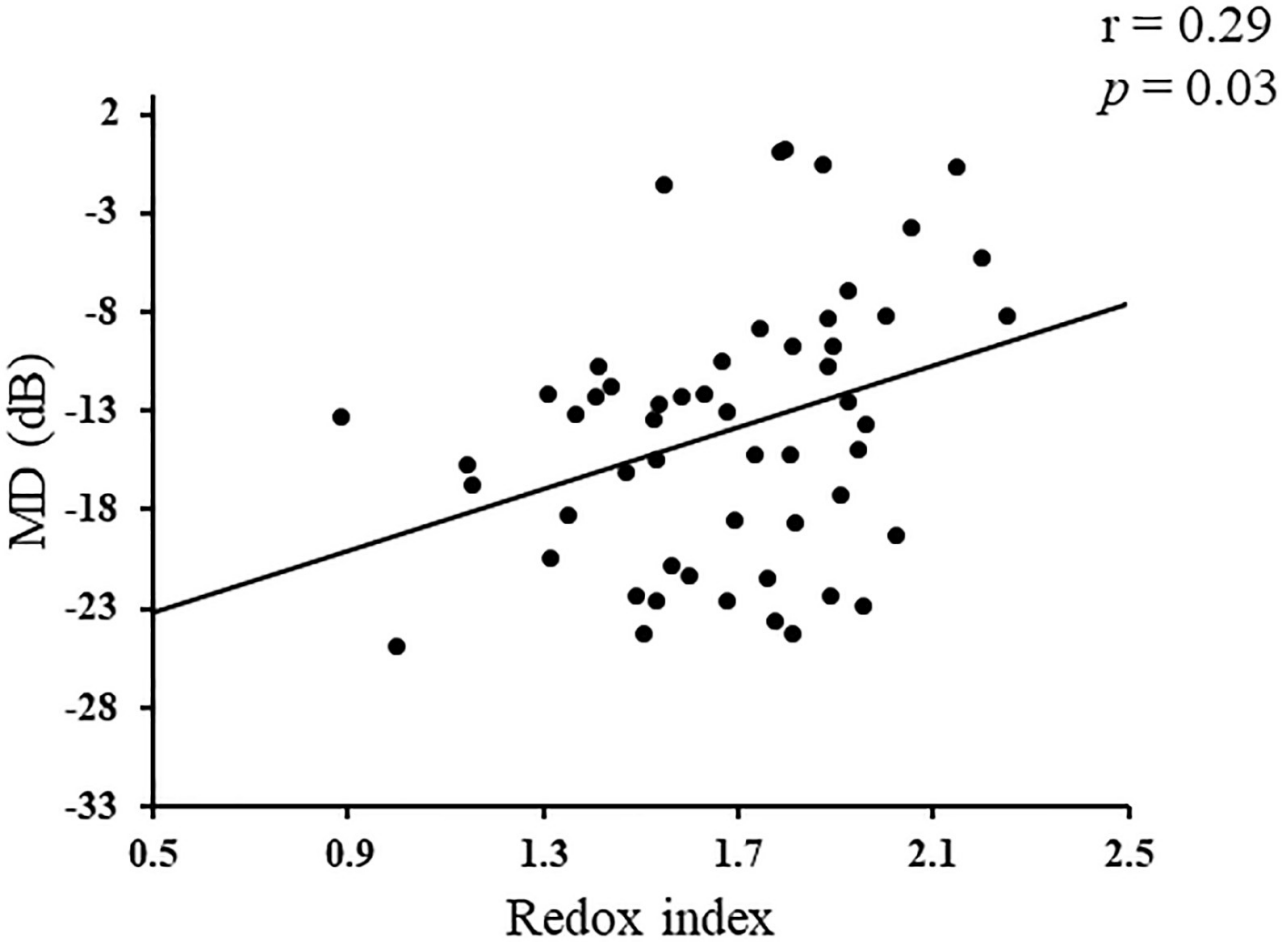
The relationship between redox index and mean deviation (MD) in patients with open angle glaucoma (OAG). Redox index correlated with MD (r = 0.29, P = 0.03, Spearman’s rank correlation test).

**Fig 4:**
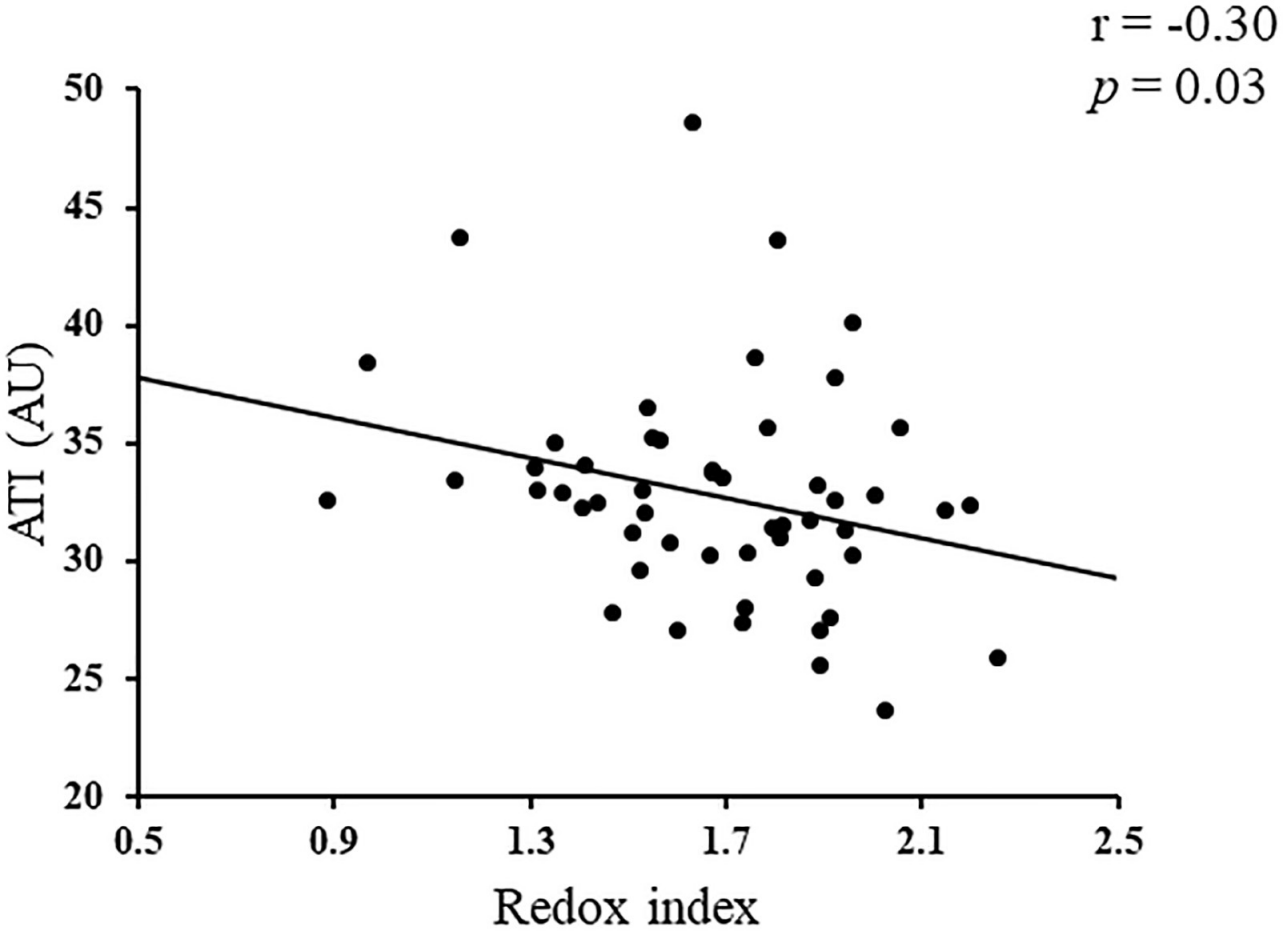
The relationship between redox index and acceleration time index (ATI) in patients with open angle glaucoma (OAG). Redox index correlated with ATI (r = 0.30, P = 0.03, Spearman’s rank correlation test). AU, arbitrary unit.

In the glaucoma group, a single regression analysis showed that redox index was significantly correlated with MD (r = 0.29, p = 0.03, Fig 3) and ATI (r = -0.30, p = 0.03, Fig 4). Redox index, however, was not significantly associated with any other clinical parameters, including age, log MAR, IOP, AL, cpRNFLT, MBR, or OPP (all p>0.05). On the other hand, there were no significant differences between GSSG level and any other clinical parameters (all p>0.05).

A multiple linear regression analysis demonstrated that cpRNFLT (β = 0.7, p<0.001) and redox index (β = 0.26, p = 0.02) were predictors of MD (Table 3). Additionally, redox index (β = -0.34, p = 0.04) was a predictor of ATI (Table 4).

**Table 3:** Multiple regression analysis of independent variables affecting mean deviation in the study groups.

Variable Dependent	Independent	β	p Value
MD (dB)	Age (years)	-0.09	0.41
	AL (mm)	-0.09	0.43
	IOP (mmHg)	0.15	0.15
	CpRNFLT (μm)	0.70	<0.001
	Redox index	0.26	0.02
	ATI (AU)	0.20	0.07

β is the standard partial regression coefficient.

MD, mean deviation; AL, axial length; IOP, intraocular pressure; CpRNFLT, circumpapillary retinal nerve fiber layer thickness; redox index, the log GSH/GSSG ratio; ATI, acceleration time index; AU, arbitrary unit.

**Table 4:** Multiple regression analysis of independent variables affecting acceleration time index in the study groups.

Variable Dependent	Independent	β	p Value
ATI (AU)	Age (years)	-0.02	0.90
	IOP (mmHg)	-0.04	0.80
	CpRNFLT (μm)	-0.34	0.13
	MD (dB)	0.41	0.09
	Redox index	-0.34	0.04

β is the standard partial regression coefficient.

ATI, acceleration time index; AU, arbitrary unit; IOP, intraocular pressure; CpRNFLT, circumpapillary retinal nerve fiber layer thickness; MD, mean deviation; Redox index, the log GSH/GSSG ratio.

**Fig 5:**
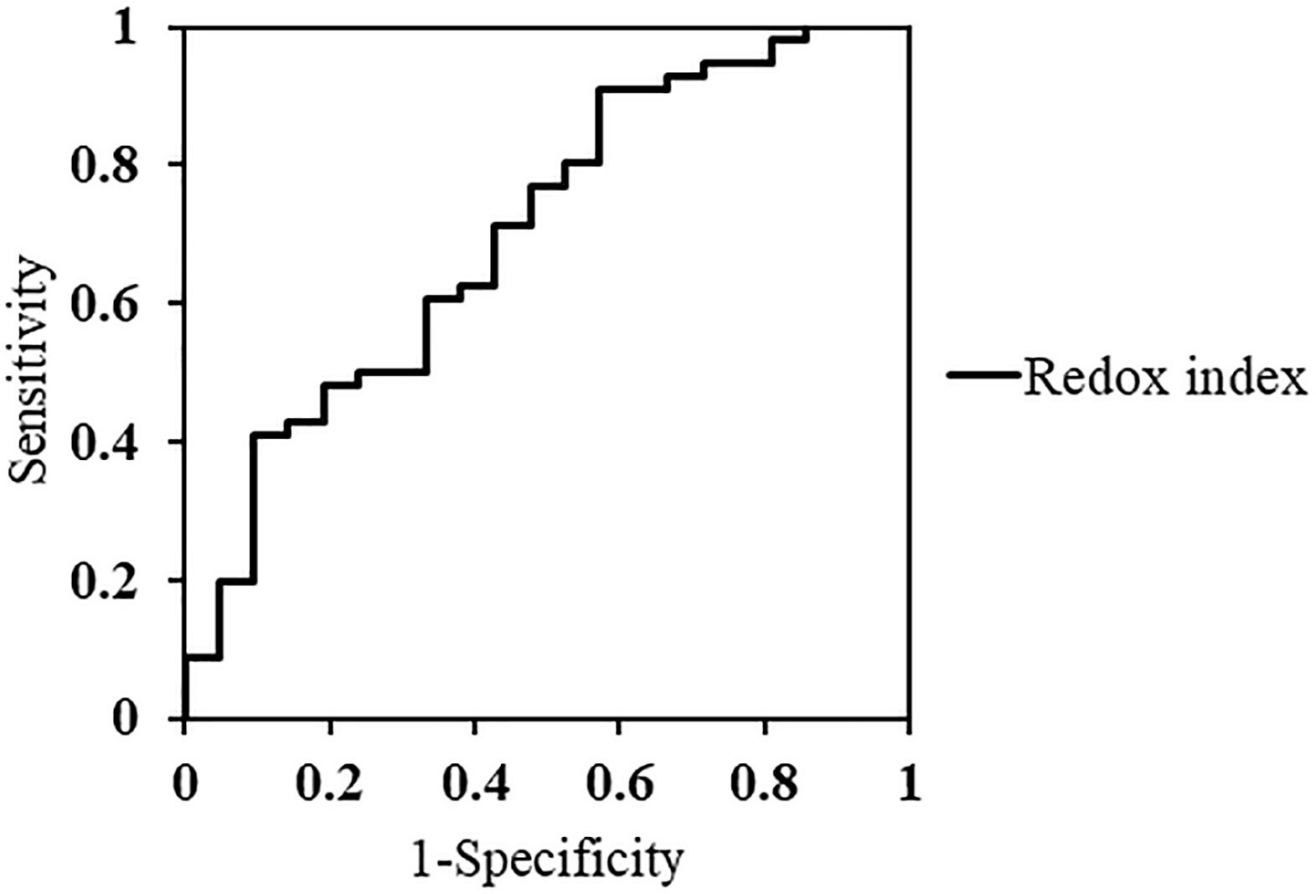
ROC curve for redox index. Receiver operating characteristic curve (ROC) curve for redox index in the study groups.

An AUC analysis revealed that redox index could differentiate between normal eyes and eyes with glaucoma (AUC; 0.70: 95% interval; 0.57–0.84). This analysis suggested that the cutoff point for redox index to maximize its sensitivity and specificity was 2.0 (sensitivity: 91.1%, specificity: 42.9%) (Fig 5).

## Discussion

In this study, we investigated GSH levels as a marker of mitochondrial function in glaucoma patients. Hamilos et al.[41] found that the concentration of t-GSH in PBMCs was 3~8 nmol/10^7^ cells; our result was remarkably similar. We found that there was an imbalance between GSH and GSSG in the PBMCs of glaucoma patients, suggesting that systemic mitochondrial dysfunction plays a distinct role in glaucoma pathology. Additionally, this study also showed that redox index was correlated with glaucoma severity (MD) and BF abnormality (ATI) in the ONH. Furthermore, multiple analyses revealed that mitochondrial function independently contributed to MD and ATI, among other clinical parameters. These findings suggest that systemic mitochondrial dysfunction could cause damage to the RGCs, and that deficient autoregulation of BF in the ONH in glaucoma may occur via impaired mitochondrial function related to redox status. In addition, the AUC analysis showed that redox index in PBMCs had the power to distinguish glaucoma eyes and control eyes and that low redox status was a specific change in glaucoma.

Abundant evidence shows that mitochondrial dysfunction, which can be induced by aging, genetics, and nutritional predispositions, plays a critical role in the pathogenesis of glaucoma.[7, 9–11, 14, 42] This includes not only focal, but also systemic mitochondrial dysfunction. Mabuchi et al. demonstrated that polymorphism of the optic atrophy 1 (OPA1) gene, which encodes a dynamin-related mitochondrial protein, was related to the pathology of OAG.[43] Additionally, we previously showed that mitochondrial DNA damage in blood cells, represented by an increased mitochondrial/nuclear DNA (mtDNA/nDNA) ratio, was associated with abnormal BF (MBR) in the ONH in glaucoma patients.[44] Taking these past results together with those of this study, it is thus reasonable to consider that systemic mitochondrial dysfunction influences hemodynamics in the ONH. However, it remains to be determined why the mtDNA/nDNA ratio, which may reflect accumulated oxidative stress, was related to blood flow volume in the ONH, while on the other hand, redox index was associated with impaired vasodilator activity. Further research is needed to solve this problem, although it has been reported that in neurodegenerative diseases, the presence of oxidative stress in PBMCs or red blood cells is associated with oxidative stress in other cells, including the RGCs, endothelial cells and astrocytes, which support neural cells and share the same micro-environment.[18, 45–49]

Previous research suggests that redox status in PBMCs could be associated with vascular endothelial function, and that altered redox status could lead to abnormal vascular autoregulation in diabetes mellitus and cardiovascular diseases.[24] [25] Endothelial cells damaged by oxidative stress reduce their production of NO, which regulates hemodynamics.[50] This change in NO action could also be related to glaucoma pathogenesis.[51] In this study, we found a relationship between low redox status in the PBMCs and vasoconstriction, as evaluated with ATI in the ONH, indicating that redox index in the PBMCs might be a biomarker of the autoregulation system, which is regulated by endothelial cells in the ONH.

Vascular dysregulation, as described above, not only damages RGCs but also activates glial cells that support the RGCs, such as astrocytes.[2, 52] Activated astrocytes change the micro-environment by releasing several compounds, such as matrix metalloproteinases (MMPs). It has been shown that upregulated MMPs in activated astrocytes in the ONH degrade the extracellular matrix (ECM) after retinal I/R injury in mice.[53] As a consequence the ECM is remodeled and degeneration of the lamina cribrosa occurs, which predisposes the RGCs towards compression[54, 55] and can cause further vascular dysregulation.[56] Indeed, we previously showed that the thickness of the lamina cribrosa, which indicates its degeneration, was related to BF in the ONH in glaucoma.[57] Astrocytes also release endothelin, which acts as a strong inducer of vasoconstriction[50] and prevents axonal transportation in the RGCs.[58] Furthermore, although NO in the astrocytes does not itself damage RGCs, it can be converted into the form of peroxynitrite (ONOO^-^). When this peroxynitrite reaches the RGCs, it can cause apoptosis if superoxide radicals (O_2_^-^) have been generated by reperfusion injury.[59] Additional evidence that peroxynitrite can cause oxidative injury comes from research in monkeys with experimental glaucoma.[60] This sequence of events promotes repeated episodes of vascular dysregulation, causing degeneration of the lamina cribrosa and repetitive unstable BF in the ONH, finally causing RGC death.[61] This mechanism would explain the hypothesized relationship between systemic mitochondrial dysfunction and MD, an indicator of visual loss, in this study. Nevertheless, we identified only one aspect of the relationship between systemic mitochondrial damage and hemodynamics, and additional work is therefore required to elucidate the overall relationship.

Although our method was quantitative and we consider that it was reasonable, there were several limitations. First, although we never collected blood from glaucoma patients sooner than 3 hours after the last meal, we did not completely standardize the condition of the subjects before blood sample collection, and factors such as diet, time when the blood test was performed, and lifestyle might have influenced the GSH concentration in the samples.[62] Second, the population of this study showed a tendency towards more severe glaucoma. Furthermore, we used control subjects with mild cataract, ERM, and dry eye rather than healthy volunteers. A comparative study between glaucoma patients and healthy volunteers will therefore also be necessary.

## Conclusion

In conclusion, our study demonstrated that mitochondrial function was lower in OAG patients than in controls. Furthermore, our findings suggest that mitochondrial dysfunction might contribute to glaucoma pathogenesis by causing vascular alterations. Increasing evidence shows that mitochondrial dysfunction plays a key role in the etiology of glaucoma: therapies targeting the mitochondria are therefore a promising approach to develop new treatments for glaucoma.

## Supporting information

S1 File Demographic data for all participants in this study. (XLSX)

S2 File Clinical parameters for OAG patients. (XLSX)
